# Notes and News

**Published:** 1887-04-23

**Authors:** 


### Notes and News.

Collected and Communicated.

Miss Eugenia Barton, late staff nurse at St. Bartholomew's Hospital, has been appointed matron to the Windsor Royal Infirmary.

Miss Julian, late lady superintendent to the General Hospital, Newark, Notts., has been appointed matron to the New Union Infirmary, Dulwich.

Hospital Sunday :—The following fixture has been made : Berwick, May 15.

Dr. Cahill, who has held the post of physician to the Berwick Dispensary and Infirmary for forty years, has resigned, Dr. Fraser being appointed to the vacancy. Dr. Cahill has been made consulting physician.

A movement is on foot for the erection of a cottage hospital at Fleetwood. Mr. Hugh C. Smith has offered to give the site and to subscribe £300 to the building fund. A fever hospital is to be built near Edinburgh, at a cost of about £1,600.

Dr. Alfred Meadows, of Poyle Manor, Bucks, died on the 19th inst., after a very short illness, at his town residence, 27, George Street, Hanover Square. The deceased was J.P. for Middlesex, and had been for many years Obstetric Physician of St. Mary's Hospital, and Lecturer on Diseases of Women at the Medical School. Dr. Meadows was lately President of the Gynecological Society, and a member of many other learned British and foreign societies. He was also author of several well-known professional works.

There is, we fear, now little probability that the Governors of Guy's Hospital will be able to raise by 1st of May the £100,000 for which they appealed. The amount at present collected is a little over £54,344, while there is a sum of £10,750 which will be given conditionally upon the amount asked for being forthcoming by 1st of May. This means that, between now and then, very nearly £35,000 must be raised. We hope that a serious effort will be made, for it would be a great pity to lose the conditional donations.

Miss Catherine Marsh has recently published a most interesting little *brochure*, telling the story of the origin of the Blackrock Convalescent Hospital, which was established during the cholera visitation in 1866. The success of Miss Marsh's exertions led her to take two houses in Brighton, where, during the past twenty years, over 9,000 patients have been treated with skill and loving care.

The annual festival dinner in aid of the funds of the Middlesex Hospital is to be held at the Langham Hotel, on Wednesday, May 18th. Lord Charles Beresford, R.N., V.C., M.P., will preside. Relying upon the liberality of the supporters of this institution, no charge will be made for the dinner.

On Easter Day a most impressive sermon was preached by the vicar, the Hon. and Rev. F. Byng, at St. Peter's, Cranley Gardens, in aid of the Brompton Hospital for Consumption. The vicar remarked that the hospital was now in urgent need of funds, while its proximity made its afflicted inmates almost their neighbours. At the close of the service a collection was made, amounting to fifty-six pounds.

On Monday, the 11th inst., a general court of the governors and supporters of the French Protestant Hospital was held at the Institution, Victoria Park, under the presidency of Mr. C. J. Shoppee, deputy governor. The little hospital is one which does a large amount of useful work among French Protestants of the East-end of London. The report, which was of a very encouraging character, was adopted. Mr. Charles Norris was elected treasurer in succession to Mr. Shoppee, and Mr. Neville Jourdain was elected hon. solicitor in the place of the late Mr. Hervé Giraud.

We have recently received the report of the seventh annual meeting of the Newton Cottage Hospital Corporation, Massachusetts, and also a copy of the *Newton Graphic* for March 5th. The latter contains a paragraph announcing a gift to the hospital of 10,000 dollars by Mrs. Elizabeth Eldridge, to be invested for the permanent benefit of the institution. A sum of 235 dollars had also been received since the publication of the annual report from Mr. Edward P. Call, as the proceeds of two theatrical performances in aid of the building fund of a new women's ward. From the report of the Corporation it appears that the hospital was only opened and dedicated on June 5th, 1886, the previous annual meetings having been held with a view to providing for its satisfactory establishment. Since that date thirty-nine patients have been treated, seventeen of whom were foreigners. Much gratitude is expressed in the report for the sympathy and support of the Ladies' Aid Association, which has contributed in no small degree to the success of the enterprise. The sum expended on building was 5,358 dollars, whilst the current expenses amounted to 1,889 dollars, and the current receipts to 3,617 dollars, thus leaving a balance in hand of 1,728 dollars. It will thus be seen that the Newton Cottage Hospital has made a good start, and we trust that it may long and successfully continue the work so well begun.

THE following vacancies are announced this week, the amount of the salary being placed in each case after the name of the institution :—

**Honorary Medical Officers.**

Chelsea Dispensary. Surgeon.
Great Northern Central Hospital. Aural Surgeon.
Infirmary for Consumption, Margaret Street, W. Visiting Physician.

**Secretary.**

Dreadnought, Seamen's Hospital, Greenwich.—£350, with commission house, etc. Age between 25 and 40.

**Resident Medical Officers.**

Birmingham Borough Asylum. Clinical Assistant. No salary.
Bristol Royal Infirmary. Assistant. Qualified.—£80.
Carmarthen Infirmary. House Surgeon. Doubly-qualified. —£100.
Chester Infirmary. Visiting Surgeon. Doubly-qualified.—£80.
City of London Hospital for Diseases of the Chest. Clinical Assistant.—£20.
Cumberland Infirmary. Assistant House Surgeon. Qualified. —£40.
Eastern Fever Hospital. Clinical Assistant.
Evelina Hospital. House Surgeon. Qualified.—£70.
Great Northern Central Hospital. Two Clinical Assistants.
Hampstead Temperance Hospital. Junior House Surgeon. Qualified.
Holborn Union. One. Fully qualified.—£100.
Kidderminster Infirmary. House Surgeon.—£140.
Liverpool Infirmary for Children. House Surgeon.—£85.
Whitechapel Union Infirmary. Assistant.—£150.
Wigan Infirmary. Junior House Surgeon. Doubly-qualified. —£80.

**Non-resident Medical Officers.**

The Hospital for Sick Children. Several Clinical Assistants.

**Matrons.**

Marylebone Infirmary. Second Assistant.—£35.
Penn Convalescent Home.—£30.

**Trained Nurses.**

Belgrave Hospital for Children. One.
Durham Hospital. One Head, £25. One for Children's Ward. —£20.
Great Northern Central Hospital. Charge Nurse.—£20.
Ipswich Hospital. Two Night.—£20.
Leeds Hospital for Women. Head.—£25.
Llanelly Hospital. One.—£25.
Newcastle Fever Hospital. One.—£25.
Rochdale Infirmary. One. Alternate day and night duty.—£22.
Royal Hospital. Waterloo Bridge Road. Surgical Nurse.—£20.
West Bromwich District Hospital. One.

---

THE following appears in the *Australian Town and Country Journal* (Sydney, New South Wales), of Feb. 19th, 1887. The modesty of "The Great English Staff of Physicians, Surgeons, and Specialists" in supporting their views is noteworthy, as such gentry are not usually so backward in coming forward. The precise address, too, "Head Office, London, England," is good, very good :—

Arrived in Sydney.

---

THE GREAT ENGLISH STAFF OF PHYSICIANS, SURGEONS, AND SPECIALISTS

have arrived at Sydney, and can be consulted until MARCH 12, at the Australian Widows' Fund Co.'s Buildings, 263, George Street. These great surgeons return every three months, and to establish a wide reputation in Sydney and N.S.W., they offer their SERVICES for this first visit FREE OF CHARGE. Let every sick person call upon the Doctors, and where no hope of recovery can be held out the patient will receive the best of advice. These Doctors have had large experience in the Hospitals of London, Edinburgh, Dublin, Paris, Vienna, Berlin, St. Petersburg, etc. To show the extent of their skill, they offer a large premium if they fail to cure any case they undertake of Consumption, Catarrh, Cancer, Nervous Debility, Dyspepsia, and Liver Complaint, Skin, Eye, and Ear Diseases, Rheumatism, Ague. Number of testimonials received in Great Britain and Europe, 29,000.

Head Office, London (Eng.).
(Established 1870.)

THE death of the Duchess of Norfolk, which occurred at Arundel Castle on the 11th inst., will cause widespread regret amongst charitable people. The Duke has always displayed the greatest interest in the welfare of charitable institutions of all descriptions, and has shown an especial desire to promote the success and welfare of hospitals. We do not remember a single occasion on which he has not consented to preside, or take part in, a hospital gathering to which he has been invited. The London hospitals especially have good cause to be grateful to his Grace, and we do but express a unanimous feeling in offering him the sincere condolences and sympathy of hospital managers everywhere.

---

MANY correspondents seem to think the editorial wisdom capable of solving any questions they may desire to have answered. Some ladies who have expressed their desire to have their names entered on the list of those willing to join a National Pension Fund, possess even a greater faith than such correspondents. For example, "One Anxious to Join" writes a long letter, but entirely omits to give either name or address, and her case is by no means an exceptional one. It is humiliating, no doubt, but we are forced to explain to these ladies, that, without their assistance, it will be impossible for us to evolve from our inner consciousness either their names or addresses, and without them, though most anxious to oblige, we find it impossible to place them on the register in question.

---

THE mistakes which the poor and ignorant are apt to make with regard to the taking of medicine at times almost pass belief, and lead us to the conclusion that the story of the good wife who applied a mustard poultice to one of her husband's *boxes* when ordered by the doctor to apply it to his *chest*, is not so wide of probability as one might at first imagine. Some years ago at the London Hospital an Irish woman presented to the porter in the out-patients' hall a ticket for medicine bearing a man's name. "Surely, my good woman," inquired the official, "you are not Terence O'Reilly?" "Shure, no, by the powers, bedad," rejoined Bridget McCarthy. "Then why do you bring this ticket with you?" continued the porter. "Shure it was on the lodging-house table, with wan or two others; but anyhow, it's the blessed physic 'll do me a world of good." The porter very rightly insisted on Bridget going back to get her right ticket, and very lucky it was for her, for, while her own ticket was found to contain a prescription for some internal malady, that of her fellow-lodger, Terence O'Reilly, was for some special ointment for a sore leg! This was an actual fact, told us by the governor of the London Hospital. But the fatal consequences of such cases of stupidity are not always so providentially averted. Quite recently, a Scotch woman, named Mary Jane McEwan, a widow, went to a Glasgow hospital, and received some medicine to be taken in doses of a tea-spoonful at a time, but instead of attending to these instructions, she drank off at once the contents of a four-ounce bottle, death ensuing in a very short time.

THE following sums have been recently received by the various Medical Charities, the name of the donor or the source from which they were obtained being given in each instance after the name of the Institution :—

**Donations.**

British Home for Incurables, Clapham. Mrs. Anderson, 10 guineas.
British Home for Incurables, Putney. Mr. A. J. Warren, £10 10*s*. In memory of the late Mrs. King, £52 10*s*.
City of London Lying-in Hospital, City Road. Clothworkers' Company, £25. Mr. J. T. Allen, 10 guineas.
Cork Street Fever Hospital, Dublin. Messrs. A. Guinness, Sons & Co., £30.
Dental Hospital of London, Leicester Square. Mr. James Packe, £26 5*s*. (second donation, making fifty guineas).
Mary Wardell Convalescent Home for Scarlet Fever, Stanmore. Hon. A. G. Tollemache, £25.
North-Eastern Hospital for Children, Hackney Road. Mr. Algernon Peckover, £50. Clothworkers' Company, £52 10*s*.
North London Hospital. Clothworkers' Company, £100. Miss Ellen Anderson, £31 10*s*. Mr. H. L. W. Lawson, M.P., £31 10*s*.
Royal United Hospital, Bath. Mr. R. D. Commans, £21.
Scarborough Hospital and Dispensary. Mr. G. W. Cattley, £20.
Sunderland Infirmary. River Wear Commissioners, £33 3*s*. 4*d*. Messrs. S. P. Austin & Son, £13 19*s*. Murton Colliery, £14 10*s*. 4*d*.
Sussex County Hospital. Mr. J. N. Winter, £26 5*s*. Mrs. J. B. Winter, £26 5*s*. Mrs. J. Shoolbred, £10 (additional).

**Legacies.**

Birmingham General Dispensary. Mr. W. Middlemore, £100.
Birmingham General Hospital. Miss Charlotte Hollins, per Chas. Hollins's Executors, £100. Mr. William Webb, £100.
Derbyshire General Infirmary. Mrs. Mary Walters, £500.
Hereford Infirmary. Right Hon. Henry Edwyn Chandos, Earl of Chesterfield, £100.
Hospital for Consumption, Brompton. Mr. W. B. Phillimore, £200.
King's College Hospital. Mr. W. B. Phillimore, £200.
Middlesex Hospital. Mr. W. B. Phillimore, £200.
Royal Hospital for Incurables, Putney. Mr. W. B. Phillimore, £200.
St. George's Hospital. Mr. W. B. Phillimore, £200.
St. Mary's Hospital, Paddington. Mr. W. B. Phillimore. £200.
University College Hospital. Mr. W. B. Phillimore, £200.

**Sale of Work.**

Paddington Green Children's Hospital. Sale of Clothing in Out-Patients' room on April 5th. £17.

**Entertainments.**

Birmingham General Dispensary. Mitchell's St. George's Football Club (Charity Match, with Prince of Wales Theatre Club), £10.
Birmingham General Hospital. Part proceeds of Charity Football Match, per Mr. H. Mitchell, Junr., £20.
Hull Royal Infirmary. Working Men's Committee in aid of the Infirmary. Proceeds of recent concert, £42.

---

THE Belvedere Fever Hospital at Glasgow, which stands in its own estate of some thirty-three acres, and is surrounded by large gardens and pleasure-grounds, was officially inspected and opened on the 4th March. It was commenced sixteen years ago, the pavilions then erected being of wood. These, however, have been gradually replaced by substantial and commodious brick pavilions, thirteen in number, having thirty-six wards in all, and capable of accommodating 390 patients. The administrative staff are located in a stone building, specially constructed for their use on the site of the old Belvedere mansion, and comfortably furnished. It contains a board-room, accommodation for the doctors, matron, and eighty-two nurses, and has sitting-rooms on each floor. The sum of £106,402 has been spent in erecting the hospital, administrative department, and boundary walls, and in laying out the grounds, whilst a further sum of £30,235 has been expended upon the small-pox hospital, which is also built on the Belvedere estate, but has a separate entrance and boundary wall of its own.

WE are indebted to Mr. J. Whitely Ward, the Treasurer, for the 1886 report of the Halifax Infirmary. The number of in-patients admitted during the year was 767, as against 732 in 1885, whilst the out-patients numbered 3,836, or 246 more than in the previous year. The income, donations and legacies to the amount of £3,245 being excluded, came to £5,796, and the current expenses reached a total of £5,323, a balance of over £470 being thus available for the uses of the current period. A noteworthy feature, and one commented upon by the Board with great satisfaction, is the success attendant upon the effort to arouse the interest of the artisans in the various mills and workshops, who have contributed nearly £1,863 to the revenue of the Infirmary during 1886. Two furnished cots in the children's ward have been presented and endowed in the course of the year, one being given by Lady Edwards in memory of the late Sir Henry Edwards, Bart., C.B., and the other by Mr. and Mrs. Hinchcliffe-Hinchcliffe, in memory of their children. Various improvements in the laundry and cooking departments are reported, and a new kitchen with the latest appliances has been added to the building. In fact, the institution seems to be in as flourishing a condition as its best friends could desire, though we cannot but note with surprise that the total cost per occupied bed, which in 1885 amounted to £48 2*s*., has increased to £54 8*s*. in 1886.

---

THE new wards of the St. Anne's Home at Bowdon, in connection with the Manchester Hospital for Consumption and Diseases of the Throat, were completed and opened for the use of patients towards the end of February. The old building, which was purchased about two years ago as an in-patient department, only afforded accommodation for some eighteen or twenty patients, and was wholly inadequate to the wants of the numerous poor people who sought admission. The two wards just added are a gift to the institution, and are fitted with ten beds each. They are constructed on the newest principles, and are each 50 ft. long by 24 ft. wide, giving a floor-space of about 120 ft., and 2,000 cubic feet of air per bed. Bath-rooms, lavatories, and rooms for the nurses are also provided. The exterior of the new building is faced with terra-cotta moulded blocks, which give a cheerful and picturesque appearance, and a covered way gives access to the older part of the establishment, and also serves as a promenade for the patients in wet weather. The chief novelty, however, is the sun-room, which, somewhat resembling a greenhouse, is placed in an angle of the building, and faces due south. It is fitted up with seats for the patients, who enter it by a staircase from the wards, and will by its means be able to enjoy the warmth and brightness of the sun, whilst duly protected from the cold and treacherous winds, which make our climate so dangerous to sufferers from lung disease. The temperature in the wards can be raised by a system of pipes, which introduce warm air underneath the skirting, whilst ventilators in the roof admit of complete change of air, which is calculated to take place about three times in the hour. A new dining-room, about 35ft. long by 18ft. wide, with large bay-windows, has also been added, and the kitchen accommodation has been increased. These arrangements give great satisfaction, and are likely to add in no small degree to the usefulness of the institution.

